# Functional and pharmacological characterization of a Shal-related K^+ ^channel subunit in Zebrafish

**DOI:** 10.1186/1472-6793-8-2

**Published:** 2008-02-08

**Authors:** Tomoe Y Nakamura, William A Coetzee

**Affiliations:** 1 Department of Molecular Physiology, National Cardiovascular Center Research Institute, Osaka, Japan; 2 Departments of Pediatrics, Pharmacology and Physiology & Neurosciences NYU School of Medicine, New York, NY, USA

## Abstract

**Background:**

K^+ ^channels are diverse; both in terms of their function and their molecular composition. Shal subunits were first described in Drosophila. There are three mammalian orthologs, which are members of the Kv4 subfamily. They are involved in neuronal firing patterns as well as control of the cardiac action potential duration.

**Results:**

Here, we report the biophysical and pharmacological characterization of zShal3, which is the ortholog of the mammalian Kv4.3 subunit, which in mammals is involved in action potential repolarization and gives rise to neuronal A-type K^+ ^currents involved in somatodendretic signal integration.

**Conclusion:**

We demonstrate that zShal has similar functional and pharmacological characteristics compared to Kv4.3 and it is similarly regulated by pharmacological agents and by the Kv4 accessory subunit, NCS-1.

## Background

*Danio rerio *is one of the few model organisms used to examine developmental regulation of vertebrate biological processes. Much has been leaned regarding transcriptional control of organogenesis, including development and growth of the heart [1] and brain [2]. Although the zebrafish is often used to study vertebrate development, random mutagenesis screens have identified several important genes involved in physiology and behavior. The importance of ion channels is exemplified by mutants such as the slow mo mutant, which disrupts the heart rate due to changes in the pacemaker current (I_h_) [3]. Another mutation in zebrafish, the lethal island beat (isl), disrupts the α_1C _L-type Ca^2+ ^channel subunit, which regulate heart growth independently of effects on contractions [4]. The role of ion transporters and Ca^2+ ^homeostasis is further emphasized by the arrhythmias observed in *tre *embryos, which have a mutation of the Na^+^-Ca^2+ ^exchanger [5]. The zebrafish may be an appropriate model for studying human inherited arrhythmias, for example in a recent study defining mutations of a delayed rectifier K^+ ^current in contributing to long QT syndrome [6]. Although the description and function of ion channels in zebrafish systems biology is beginning to emerge [7-11], little is known about the expression and functional characteristics of most zebrafish ion channels.

In Drosophila, *Shaker *was the first type of K^+ ^channel to be described [12]. This was soon followed by homology cloning of *Shab, Shaw and Shal *[13]. Many more voltage-activated K^+ ^channels have since been described in mammals [14], but the corresponding preferential mammalian nomenclature for these first four are Kv1, Kv2, Kv3 and Kv4 [15], with several members in each of these subfamilies. The Kv4 subfamily contains three members; Kv4.1, Kv4.2 and Kv4.3 [16]. The Kv4.x K^+ ^channels are expressed in a variety of tissue, with particularly high levels in the brain and heart. The Kv4 subunits form functional channels, with very similar biophysical and pharmacological properties, that are responsible for transient, voltage-dependent K^+ ^currents in the nervous system (A currents) in the neuronal system. These somatodendritic subthreshold A-type K^+ ^current in nerve cells contribute significantly to determine somatodendritic signal integration. The corresponding K^+ ^current in the heart is called the transient outward K^+ ^current (I_to_), which contributes to action potential repolarization [17].

Shal-type K^+ ^channel subunits are highly conserved evolutionary and are present even in primitive metazoans such as the jellyfish [18]. We show using sequence and phylogenetic analysis that orthologs of all three mammalian Kv4 subfamily members exist in *Danio rerio*. We collectively named these subunits zShals, in keeping with the nomenclature where "Kv" is reserved for mammalian subunits [15]. We also describe the biophysical and pharmacological properties of zShal3 (the ortholog of the mammalian Kv4.3), as well as its regulation by the Kv4 accessory subunit, NCS-1. Characterization of channel function and distribution is an important first step in understanding that the physiological role of ion channels in this model animal system.

## Methods

### Electrophysiological measurements in oocytes

Stage V-VI oocytes were prepared from *Xenopus laevis *and 50 nl of in vitro transcribed cRNA (0.1–18 ng) were injected using a 10 μl micropipette (Drummond Scientific Co, Broomall, PA). Two to three days after the injection, oocytes were voltage-clamped using the standard two-electrode voltage-clamp technique as described previously [19]. Recordings were obtained using a Geneclamp 500 amplifier (Axon Instruments, Inc.) with data sampled at 5 kHz and filtered at 1 kHz. Currents were elicited by depolarizing steps from -100 to +60 mV in 10 mV increments every 15 s from a holding potential of -120 mV (to allow full recovery from inactivation). The recording chamber was continually perfused (1 ml/min). To avoid contamination with Ca^2+^-activated Cl^- ^currents, a low Cl^- ^recording solution containing in (mM) Na-glutamate 96, K-glutamate 2, CaCl_2 _1.8, MgCl_2 _1, HEPES 5 (pH 7.5 adjusted with NaOH) was used. All experiments were performed at room temperature (20 ± 2°C).

### Data analysis

Data were analyzed using the pClamp suite of software (Axon Instruments) and Origin for Windows (Microcal Software). Leak subtraction was not performed.

#### Inactivation parameters

the time constants of inactivation were obtained by fitting the current traces to a sum of two exponential functions.

#### Recovery from inactivation

A double pulse protocol was used to access the time course of recovery from inactivation. Two depolarizing pulses to +20 mV were separated by intervals of increasing duration (from 20 to 460 ms in 40 ms increments) at -120 mV. The peak current amplitude during the test pulse (I) was normalized to that recorded during the first pulse (I_o_) and plotted as a function of the inter-pulse duration. Curve fitting of the data points was performed to $\frac{I}{I_{o}}=I_{\max}+A\cdot e^{\frac{-t}{\tau }}$, where I_max _is the maximum current during the recovery time (not restrained to 1). A and τ are the relative amplitude and time constant respectively.

#### Steady-state inactivation

A pre-pulse voltage protocol was used. The membrane was held at voltages ranging between -150 mV and -10 mV for a period of 10 seconds (to obtain the complete steady-state inactivation) followed by a 1 s test pulse to +20 mV, during which peak current amplitude (I) was measured. Data were normalized to the peak current amplitude following a pre-pulse at -150 mV (I_0_), and plotted as a function of pre-pulse potential. The line through the data represents the best nonlinear least-squares fit to a Boltzmann function, $\frac{I}{I_{o}}=\frac{1}{\left(1+e^{\left(\frac{V-V_{h}}{k}\right)}\right)}$, where V_h _is the voltage where half-maximal inactivation was observed and k is the slope factor.

#### Normalized conductance-voltage curve

Conductance (G) was calculated with the following equation: G = I_peak_/(E-E_K_), where I_peak _is the peak current amplitude, E is the test potential and E_K _is the equilibrium potential for K^+ ^(calculated to be -99 mV under our experimental conditions). The conductance (G) was normalized to the maximal conductance at +60 mV (G_max_) and plotted as a function of the test potential.

#### Statistics

Comparisons between two groups were performed using the Student's t-test or the Rank Sum test. To test for overall significance when using more than two groups, we used the 1 way-ANOVA or, when tests for normality failed, the non-parametric Kruskall-Wallis test (Sigma Stat, Jandel Scientific). If the overall significance was obtained, multiple comparisons were performed to a control group respectively using the Dunnet's t-test or Dunn's test. Values of p < 0.05 were considered statistically significant.

## Results

### Identification of zShal3

**Figure 1:**
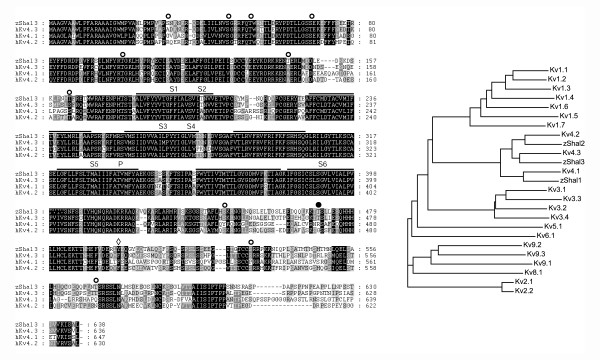
Comparison of zShals and mammalian Kv4s. **A**: Alignment of *Danio rerio *zShal3 amino acid sequence (NM_199802.1) with those of human Kv4.1 (NP_004970), human Kv4.2 (NP_036413) and the short splice variant of human Kv4.3 (AAF01045). Putative transmembrane domains and the pore region are indicated by horizontal bars. Potential PKC phosphorylation sites are indicated by open symbols, a potential PKA phosphorylation site is indicted by a solid symbol and a putative tyrosine phosphorylation site by a diamond. The T1 (or K^+ ^channel tetramerisation domain) is a N-terminal, cytoplasmic tetramerisation domain of voltage-gated K^+ ^channels encodes molecular determinants for subfamily-specific assembly of alpha-subunits into functional tetrameric channels. This domain is present between amino acids 42 to 131 of zShal3 (IILNVS ... PEIISDC). zShal3 also contains the K^+ ^channel signature sequence, which is a highly conserved stretch of amino acids (G- [YF]-G.-D) in the P-region. **B**: Phylogenetic tree comparing zShals with representative mammalian Kv channel subunits. The mammalian (all human) sequences used were Kv4.1 (NP_004970), Kv4.2 (NP_036413) and Kv4.3 (AAF01045): Multiple sequence alignment, tree bootstrapping and tree generation was performed using ClustalW.

Homology searches of the zebrafish EST databases [20] revealed an entry (fj34e05) with sequence similarity with the mammalian Shal-related subunit, Kv4.3. We tentatively named this subunit zShal3. We obtained the cDNA and sequencing revealed the full-length zShal3 coding region, in addition to some 5'- and 3'- untranslated regions (the nucleotide sequence of zShal3 was recently deposited in GenBan, NM_199802). The coding region is located on chromosome 24 [Ensembl entry AL929168.3.2001-50304, genomic location 8886207 to 8887319 (-)]. When translated, the amino-acid sequence of zShal3 is very similar to the mammalian Kv4's. The sequence identity is respectively 77%, 73% and 65% with human Kv4.3, Kv4.2 and Kv 4.1 subunits (Fig 1). Most divergence occurs in the intracellular N- and C-termini. Important structural features of the mammalian Kv4 subunits are conserved, including a six-transmembrane topology, a T1 domain and a K^+ ^channel signature sequence. zShal3 also bears several putative consensus sequences for phosphorylation by PKC, as well as a single consensus sequence each for PKA and tyrosine phosphorylation (Fig 1).

zShal3 corresponds to the short splice variant of mammalian Kv4.3. The long splice variant contains the sequence GLSYLVDDPLLSVRTSTIK within the intracellular C-terminus. We did not identify a genomic region on this zebrafish chromosome that translates to a similar sequence and it is unclear at this point whether zebrafish also generates two zShal3 splice variants.

### Biophysical characterization of zShal3

**Figure 2:**
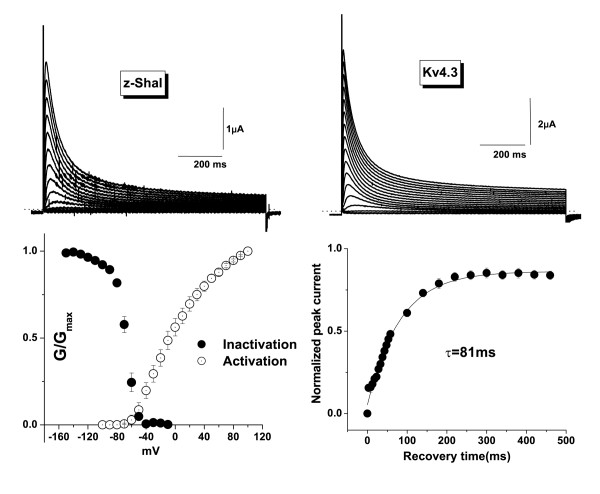
Electrophysiological properties of zShal3 expressed in *Xenopus *oocytes. **A and B: **Representative traces of zShal3 (A) and Kv4.3 currents (B). Currents were elicited by 1000 ms voltage steps from a holding potential of -120 mV to test potentials between -100 and +50 mV in 10 mV increments at every 15 s.**C: **Voltage dependence of activation and inactivation of zShal3 currents. The conductance-voltage curves were constructed by dividing the current by the driving force and normalizing to the maximal conductance (G_max_) at +120 mV for activation and at -150 mV for inactivation curve.**D: **Recovery from inactivation of zShal3 currents at -120 mV.

We injected *Xenopus *oocytes with zShal3 cRNA and recorded currents 2–4 days post-injection. Macroscopic two-electrode voltage clamp currents resembled those of Kv4.3-injected oocytes. Upon depolarization zShal3 currents activated rapidly and inactivated with maintained depolarization (Fig 2). The inactivation time course is similar to that of mammalian Kv4.3 The time constants of fast and slow inactivation at +60 mV for zShal3 and Kv4.3 were, τ_fast _= 46.7 ± 0.2 ms (85.7% of contribution) and 45.6 ± 0.11 ms (86.3% of contribution), τ_slow _= 256 ± 2.1 ms and 258 ± 1.3 ms, respectively (n = 6 for each group, P = NS). zShal3 currents activated with depolarizations beyond -70 mV. The voltage at which half-maximal steady-state inactivation is achieved is -69 ± 0.7 mV. Recovery from inactivation was determined using a two-step voltage protocol and the time constant was 81 ms at -120 mV.

### Pharmacological blockade of zShal3 currents

**Figure 3:**
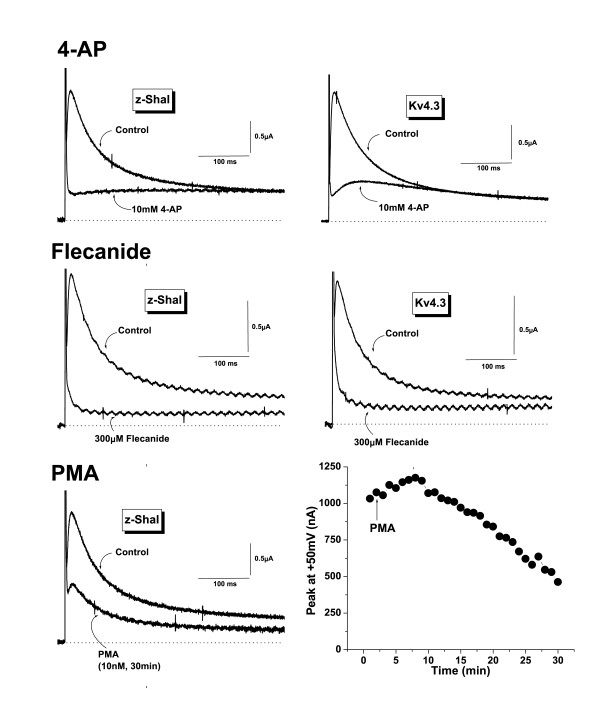
Pharmacological characterization of z-Shal3 currents. **A and B: **Effect of 4-aminopyridine (4-AP, 10 mM)(A) and flecanide (300 μM) on zShal3 (left) and Kv4.3 currents (right). **C and D: **Effects of PKC activation on zShal3 currents. zShal3 currents before and 30 min after exposure to 10 nM PMA (C). Currents were elicited by 900 ms voltage steps from a holding potential of -120 mV to the test potential at +50 mV. Time course of the effect of PMA (10 nM) on zShal currents (D). The peak current amplitude was normalized to the current amplitude before PMA application (time = 0). Results are expressed as mean ± SEM of 8 experiments in each group.

Kv4 currents are typically blocked by compounds such as 4-aminopyridine (4-AP) and flecainide [21-23]. A comparison of effects of these compounds on Kv4.3 and zShal3 is shown in Fig 3. Both currents were effectively blocked by high 4-AP concentrations (10 mM in this example). At low concentrations (50 μM), 4-AP does not block Kv4 currents (not shown). Flecainide (30 μM) also effectively blocked both Kv4.3 and zShal3 currents. These data demonstrate that structural elements required for channel block by these compounds are conserved between mammals and fish. In *Xenopus *oocytes, we previously showed that Kv4.3 currents are time-dependently blocked by phorbol esters [19]. Activating PKC with PMA (10 nM) led to a similar progressive inhibition of zShal currents (Fig 3C). Interestingly, before inhibition occurred, we consistently saw a slight activation of zShal currents. This was also often observed with mammalian Kv4s (not shown).

### Modification of zShal3 by accessory subunits

**Figure 4:**
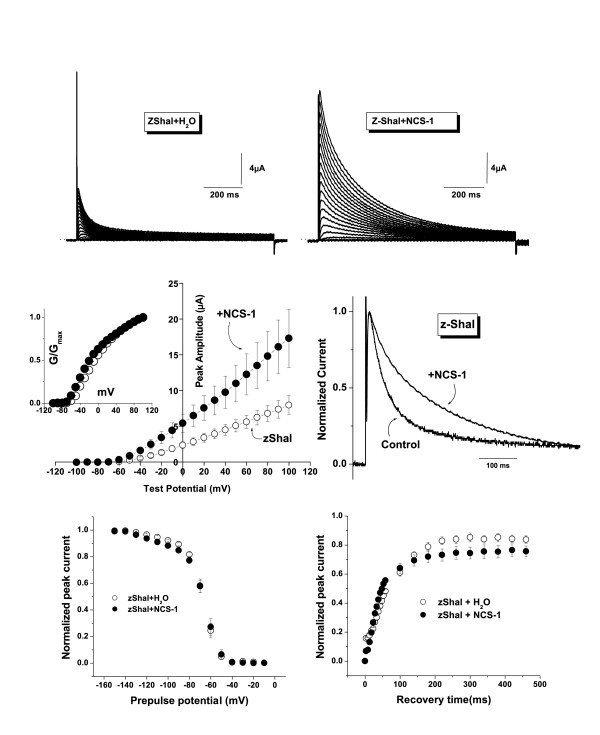
Effects of NCS-1 on zShal3 currents. **A and B: **Representative traces of zShal3 currents expressed with or without NCS-1.**C: **Averaged current-voltage (I-V) relationship of peak zShal3 current with (●) or without (○) NCS-1. The inset depicts the conductance-voltage curves that were constructed by dividing the current by the driving force and normalizing to the maximal conductance (G_max_) at +100 mV.**D: **zShal3 and zShal3/NCS-1 currents, normalized to the maximal peak amplitudes at +50 mV, to illustrate the effects of NCS-1 on the inactivation time course.**E: **Voltage dependence of steady-state inactivation for zShal3 (○) and zShal3/NCS-1 currents (●)(n = 8 for each experiment).**F: **Time course of the recovery from inactivation for zShal3 (○) and zShal3/NCS-1 currents (●)(n = 8 for each experiment).

In mammalian heterologous expression systems and in *Xenopus *oocytes, Kv4 current amplitudes are increased by co-expression with the accessory proteins, KChIPs and NCS-1. These accessory subunits additionally slow the inactivation time course [24-26]. We examined the effects of NCS-1 (also called frequenin) on zShal3 currents. As with Kv4 currents, zShal3 currents were enhanced in oocytes co-injected with NCS-1 cRNA (Fig 4A &4B). The peak zShal3 currents with and without NCS-1 were 11.0 ± 2.6 and 5.1 ± 0.8 μA at +50 mV, respectively, (Fig 4A &4B). The increase in zShal3 currents occurred at all test potentials examined without a significant shift in voltage dependence of activation as shown in the normalized conductance-voltage relationships (Fig. 4C & inset). NCS-1 also slowed the time course of zShal3 inactivation (Fig 4D). The time constants of fast and slow inactivation at +60 mV for zShal3 currents with and without NCS-1 were τ_fast _= 105 ± 2.0 ms (60.3% of contribution) and 38.7 ± 0.2 ms (89.7% of contribution), τ_slow _= 377 ± 12 ms and 243 ± 3.6 ms, respectively (n = 8 for each group, P < 0.05). As observed for Kv4 channels [25], there was no effect on the voltage-dependence of inactivation. The voltage at which half-maximal steady-state inactivation occurred (V_1/2_) were -69.1 ± 0.7 mV and -68.4 ± 1.1 mV for zShal3 and zShal3/NCS-1 currents, respectively and the slope factors (k) were 7.9 ± 0.6 and 9.8 ± 1.0 respectively; n = 8 for each experiment; Fig 4E). A small but significant acceleration of recovery from inactivation was induced by NCS-1 (time constants of recovery from inactivation (τ) were 45.7 ms and 81.4 ms with and without NCS-1 respectively; Fig 4F).

## Discussion

### Comparison with mammalian Kv4 subunits

We identified a zebrafish homolog of the mammalian Kv4.3 subunit, which we named zShal3. It has a high degree of sequence similarity (65–77% identity) with members of the Kv4 subfamily. Within the transmembrane domains (S1 to S6) and pore region the sequence similarity is much higher (>90%), suggesting evolutionary conservation of these regions responsible for channel function. The three mammalian Kv4 subunits differ from each other in defined regions of the intracellular N- and C-termini. It is interesting that most of the sequence divergence between zShal3 and Kv4's also occur in these regions, suggestive of subunit-specific roles of these divergent regions.

### Biophysical and pharmacological properties of zShal3

zShal3 behaved in most respects identically to mammalian Kv4.3 channels when expressed in *Xenopus *oocytes. The "threshold" voltage for activation is around -70 mV, which is similar to Kv4.3 when expressed in *Xenopus *oocytes [27]. This is not entirely surprising given the sequence similarity on the core region (S1 to S6) of the polypeptides. Once activated, zShal3 inactivates at rates similar to Kv4.3. The molecular mechanisms involved in Kv4 inactivation are not well understood, but classic N-type ("ball and chain") or C-type (pore-collapse) inactivation may not occur. There may be a concerted interaction between the intracellular N- and C-termini in causing Kv4.1 inactivation [28] and it is likely that a similar mechanism may operate to cause zShal3 inactivation. The similarities in voltage-dependence of inactivation and rates of recovery from inactivation further support the notion of evolutionary conserved inactivation mechanisms. The zShal subunit may be useful in further elucidating Kv4 inactivation, which is thought to occur predominantly from a pre-open closed state [29]. Pharmacologically, both zShal3 and Kv4.3 are blocked by 4-AP and flecainide. There is evidence that the 4-AP binding site is on the cytoplasmic surface of the Kv4.2 channel at, or adjacent to, the domains involved in channel inactivation [21,29]. Flecainide sensitivity, in contrast, is mediated by a single residue in the S6 transmembrane domain of voltage-gated potassium channels [30]. This residue is L392 in the case of Kv4.2, which is conserved in zShal3 (and also in the other two zShals; see later). Because of this sequence conservation, it is likely that these compounds may also block zShal3 currents by interaction with these homologous residues. Furthermore, as observed for Kv4.3 current [19], zShal3 current was also largely modulated by PKC activation, suggesting that a similar functional role for signal transduction-mediated modulation of A-type current may exist in zebrafish.

### Modulation of zShal3 by accessory subunits

KChIPs and NCS-1 (frequenin) strongly modulate Kv4 currents by increasing their expression levels and slowing their inactivation [24-26]. Since NCS-1 may be the evolutionary ancestor of the Ca^2+^-binding protein family[31], we examined the effect of NCS-1 and found this Kv4 accessory protein to affect zShal3 in an identical manner. KChIPs (and likely also NCS-1) bind to Kv4 subunits by interacting with specific residues. A major interaction site for KChIPs binding within Kv4.2 lies within the N-terminus – particularly with the proximal N-terminal region [24,32,33]. The absolute conservation of residues and domains involved in interaction, despite divergence in other parts of the N-terminus, suggests an evolutionary conserved mechanism of modulation of Shal (Kv4) subunits by accessory subunits, which predates the vertebrate evolutionary branching point of mammals and fish. Indeed, inspection of zebrafish genome suggests orthologs of at least some of the KChIPs (zKChIPs, Genbank entries AAH96914, XP_687972 and XP_694748), as well as a NCS-1 ortholog (AAO34710). It remains to be seen whether these zKChIPs or zNCS-1 will have any influence on zShal currents.

### Other Shal-related family members in zebrafish

There are three members in the mammalian Kv4 subfamily (Kv4.1, Kv4.2 and Kv4.3). Similarity searches of the zebrafish genome revealed homologs for each of these mammalian Kv4 subunits. The putative subunits zShal1, zShal2 and zShal3 are respectively located on zebrafish chromosomes 19 (Ensembl ID: ENSDART00000009506), 4 (ENSDART00000042673) and 24 (Zv5_scaffold1202.6) and correspond to Kv4.1, Kv4.2 and Kv4.3. The sequence similarity between zShals and Kv4's are greater than observed with other mammalian Kv subunits (Fig 1). Given this high degree of sequence similarity, as well as the similarities in the functional and pharmacological properties between zShal3 and mammalian Kv4's (this study), it is expected that these additional zShal members will have biophysical and pharmacological properties resembling their mammalian orthologs. However, this prediction (as well as their relative tissue-distributions) still needs to be examined.

### Possible functional significance of zShal expression in Zebrafish

Without detailed electrophysiological recordings of native currents in Zebrafish or in the absence of knockout experiments, it is not possible to determine the functional significance of zShal expression in the fish at this point. Given the similarities in function and regulation of zShal to their mammalian orthologs (Kv4 subunits), one would expect these channels to have similar functions in the fish as they do in the mammal. Known functions of Kv4 subunits in mammals include a role in somatodendritic subthreshold A-type K^+ ^currents, which operate in the subthreshold range of membrane potentials [16] and may be involved in determining the properties of dendritic, backpropagating action potentials and long-term potentiation [34]. Preliminary whole-mount in-situ hybridization experiments suggest that zShal3 may be expressed in neural tissues of Zebrafish embryos (data not shown). Depending on the protein distribution and dentritic localization of zShals, they may have similar functions. If any of the zShals (see above) are expressed in the Zebrafish heart, then by analogy with the mammalian heart, one would expect these subunits to give rise to a 'transient outward K^+ ^current', which helps to determine action potential shape and cardiac contractile properties [35]. The regulation of zShals by accessory proteins such as NCS-1 (or possibly by KChIP orthologs) also suggests a similarity in how these channels are regulated in-vivo.

## Conclusion

We identified a member of the Shal subfamily of K^+ ^channel subunits in Zebrafish and named it zShal3, since it appears to be the ortholog of the mammalian Kv4.3 subunit. We report the biophysical and pharmacological characterization of zShal3. We demonstrate that zShal has similar functional and pharmacological characteristics compared to Kv4.3 and it is similarly regulated by pharmacological agents and by the Kv4 accessory subunit, NCS-1.

## Authors' contributions

TYN was responsible for the conduct of experiments. WAC was responsible for the study design. TYN and WAC performed data analysis, presentation and manuscript preparation. Both authors read and approved the final manuscript.
